# Supplementary material for: Empirical distributions of time intervals between COVID-19 cases and more severe outcomes in Scotland
Source: PLoS One. 2023 Aug 16;18(8):e0287397. doi: 10.1371/journal.pone.0287397 (PMC10431635; doi:10.1371/journal.pone.0287397)
Supplement: S1 Table — These are specified by age, sex and the relative deprivation of residing DZ (equally dividing the DZs into three groups, based on the overall rank in the Scottish Index of Multiple Deprivation). (PDF) [file pone.0287397.s001.pdf]

S1 Table

Empirical distributions of time intervals between COVID-19 cases and more severe outcomes in Scotland

Anthony J Wood, Rowland R Kao

Age range		Total	Total linked	Same-day events	Mean (d)	St. dev. (d)	Median (d)	[5%, 95%]
Δt_{CH}	20–49	6895	5866 (85%)	936 (16%)	7.41	4.22	7	[1, 15]
	50–59	5042	4243 (84%)	820 (19%)	7.24	4.13	7	[1, 15]
	60–69	5358	4292 (80%)	1267 (30%)	6.80	4.27	6	[1, 15]
	70+	14429	9743 (68%)	3814 (39%)	6.48	4.68	6	[1, 16]
	Total	31724	24144 (76%)	6837 (28%)	6.95	4.39	6	[1, 15]
Δt_{CI}	20–49	868	748 (86%)	72 (10%)	7.70	4.17	7	[1, 15]
	50–59	918	817 (89%)	85 (10%)	7.64	4.01	7	[1, 14]
	60–69	990	909 (92%)	103 (11%)	7.05	4.16	7	[1, 15]
	70+	738	642 (87%)	77 (12%)	6.79	4.32	6	[1, 15]
	Total	3514	3116 (89%)	337 (11%)	7.31	4.17	7	[1, 15]
Δt_{CM}	20–49	234	179 (76%)	<10 (2%)	12.59	7.16	12	[2, 25]
	50–59	513	426 (83%)	<10 (1%)	12.27	7.00	11	[2, 26]
	60–69	1106	895 (81%)	<10 (1%)	12.59	7.10	12	[2, 25]
	70+	6453	5407 (84%)	48 (1%)	11.79	6.38	11	[3, 24]
	Total	8306	6907 (83%)	64 (1%)	11.94	6.54	11	[2, 24]

Sex		Total	Total linked	Same-day events	Mean (d)	St. dev. (d)	Median (d)	[5%, 95%]
Δt_{CH}	Female	15498	11878 (77%)	3237 (27%)	6.95	4.42	6	[1, 15]
	Male	16226	12266 (76%)	3600 (29%)	6.95	4.36	6	[1, 15]
	Total	31724	24144 (76%)	6837 (28%)	6.95	4.39	6	[1, 15]
Δt_{CI}	Female	1330	1202 (90%)	116 (10%)	7.30	4.22	7	[1, 15]
	Male	2184	1914 (88%)	221 (12%)	7.32	4.14	7	[1, 15]
	Total	3514	3116 (89%)	337 (11%)	7.31	4.17	7	[1, 15]
Δt_{CM}	Female	3890	3246 (83%)	26 (1%)	11.93	6.56	11	[2, 24]
	Male	4416	3661 (83%)	38 (1%)	11.96	6.53	11	[2, 24]
	Total	8306	6907 (83%)	64 (1%)	11.94	6.54	11	[2, 24]

Deprivation band		Total	Total linked	Same-day events	Mean (d)	St. dev. (d)	Median (d)	[5%, 95%]
Δt_{CH}	1 (High)	14952	11560 (77%)	3484 (30%)	6.82	4.40	6	[1, 15]
	2 (Medium)	9416	7145 (76%)	1979 (28%)	6.98	4.36	6	[1, 15]
	3 (Low)	7356	5439 (74%)	1374 (25%)	7.17	4.40	7	[1, 15]
	Total	31724	24144 (76%)	6837 (28%)	6.95	4.39	6	[1, 15]
Δt_{CI}	1 (High)	1719	1515 (88%)	187 (12%)	7.13	4.13	7	[1, 15]
	2 (Medium)	1051	938 (89%)	89 (9%)	7.37	4.24	7	[1, 15]
	3 (Low)	744	663 (89%)	61 (9%)	7.64	4.15	7	[1, 15]
	Total	3514	3116 (89%)	337 (11%)	7.31	4.17	7	[1, 15]
Δt_{CM}	1 (High)	3773	3120 (83%)	27 (1%)	11.76	6.52	11	[2, 24]
	2 (Medium)	2522	2113 (84%)	23 (1%)	12.02	6.51	11	[2, 24]
	3 (Low)	2011	1674 (83%)	14 (1%)	12.19	6.62	11	[3, 25]
	Total	8306	6907 (83%)	64 (1%)	11.94	6.54	11	[2, 24]

Summary statistics of case intervals Δt. These are the recorded times between registered COVID-19 (C)ases, and more severe outcomes: (H)ospital admission Δt_{CH}, (I)CU admission Δt_{CI}, and (M)ortality Δt_{CM}. These are differentiated by age range (top), patient sex (middle), and the SIMD deprivation band of the individual's residing datazone (bottom). *Total*: Number of that outcome found in the eDRIS data. *Total linked*: Number of that outcome found with an associated linked case. *Same-day events*: Number of linked events with where the case was reported with the same date. *Mean/median interval*: Of the linked events found, the mean/median time interval between case and outcome. *St. dev.*: The standard deviation of the intervals. *[5%, 95%]*: The values that bound the central 90% of intervals. The mean, median, standard deviation and [5%, 95%] calculations all exclude same-day events.
