# Supplementary material for: Empirical distributions of time intervals between COVID-19 cases and more severe outcomes in Scotland
Source: PLoS One. 2023 Aug 16;18(8):e0287397. doi: 10.1371/journal.pone.0287397 (PMC10431635; doi:10.1371/journal.pone.0287397)
Supplement: S2 Table — These are specified by the same groups as the case intervals. (PDF) [file pone.0287397.s002.pdf]

S2 Table

Empirical distributions of time intervals between COVID-19 cases and more severe outcomes in Scotland

Anthony J Wood, Rowland R Kao

Age range		Total linked	Mean (d)	St. dev. (d)	Median (d)	[5%, 95%]
Δt_{HI}	20–49	696	1.54	2.74	0	[0, 6]
	50–59	807	1.92	3.12	1	[0, 8]
	60–69	877	2.17	3.32	1	[0, 9]
	70+	623	3.01	3.99	1	[0, 11]
	Total	3003	2.13	3.34	1	[0, 9]
Δt_{HM}	20–49	97	10.18	6.58	10	[1, 20]
	50–59	275	9.45	5.82	9	[1, 19]
	60–69	632	10.16	5.88	10	[2, 20]
	70+	3364	9.51	5.56	9	[1, 19]
	Total	4368	9.61	5.65	9	[1, 20]
Δt_{IM}	20–49	64	9.39	5.86	10	[1, 18]
	50–59	147	10.06	5.77	10	[2, 19]
	60–69	301	9.82	6.08	9	[1, 20]
	70+	357	7.55	5.29	7	[1, 17]
	Total	869	8.90	5.80	8	[1, 19]
Sex		Total linked	Mean (d)	St. dev. (d)	Median (d)	[5%, 95%]
Δt_{HI}	Female	1120	2.06	3.26	1	[0, 9]
	Male	1883	2.18	3.38	1	[0, 9]
	Total	3003	2.13	3.34	1	[0, 9]
Δt_{HM}	Female	1957	9.25	5.73	8	[1, 20]
	Male	2411	9.91	5.57	10	[1, 20]
	Total	4368	9.61	5.65	9	[1, 20]
Δt_{IM}	Female	295	8.28	5.86	7	[1, 19]
	Male	574	9.22	5.74	9	[1, 19]
	Total	869	8.90	5.80	8	[1, 19]
Deprivation band		Total linked	Mean (d)	St. dev. (d)	Median (d)	[5%, 95%]
Δt_{HI}	1 (High)	1479	1.95	3.14	1	[0, 8]
	2 (Medium)	900	2.29	3.50	1	[0, 9]
	3 (Low)	624	2.33	3.53	1	[0, 10]
	Total	3003	2.13	3.34	1	[0, 9]
Δt_{HM}	1 (High)	2118	9.32	5.59	9	[1, 19]
	2 (Medium)	1338	9.69	5.70	9	[1, 20]
	3 (Low)	912	10.19	5.65	10	[2, 20]
	Total	4368	9.61	5.65	9	[1, 20]
Δt_{IM}	1 (High)	417	8.67	5.66	8	[1, 19]
	2 (Medium)	268	9.40	6.05	9	[1, 19]
	3 (Low)	184	8.68	5.70	8	[1, 19]
	Total	869	8.90	5.80	8	[1, 19]

Summary statistics of nosocomial intervals. These are the recorded intervals between severe COVID-19 outcomes: between (H)ospital admission and (I)CU admission Δt_{HI}, between hospital admission and (M)ortality Δt_{HM}, and between ICU admission and mortality Δt_{IM}. These are differentiated by age range (top), patient sex (middle), and the SIMD deprivation band of the individual's residing datazone (bottom). *Total linked*: Number of that outcome found with an associated prior event in the period studied. *Mean/Median interval*: Of the linked events found, the mean/median time interval between the two outcomes. *St. dev.*: The standard deviation of the intervals. *[5%, 95%]*: Bounding the central 90% of intervals.
