# Supplementary material for: Empirical distributions of time intervals between COVID-19 cases and more severe outcomes in Scotland
Source: PLoS One. 2023 Aug 16;18(8):e0287397. doi: 10.1371/journal.pone.0287397 (PMC10431635; doi:10.1371/journal.pone.0287397)
Supplement: S1 File — (PDF) [file pone.0287397.s003.pdf]

S1 Figures

Empirical distributions of time intervals between COVID-19 cases and more severe outcomes in Scotland

Anthony J Wood, Rowland R Kao

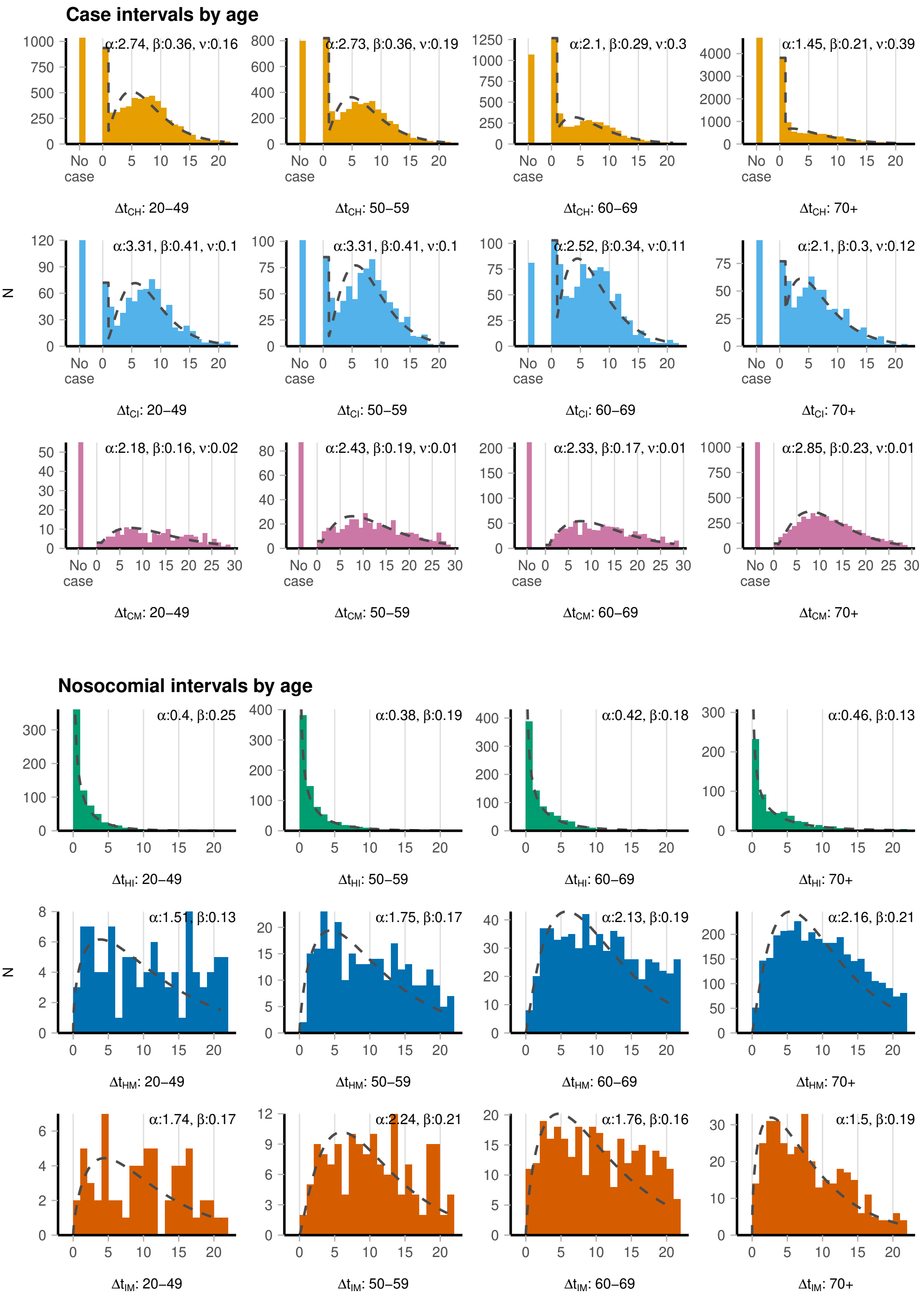

Figure 1: Interval distributions, broken down by age group. As with the aggregated distribution, a "no-case" entry is logged in the case intervals when a severe COVID-19 outcome (such as a hospitalisation) is identified, but has no associated case. α, β, are fit values for the gamma distribution rate, shape and ν is the zero inflation parameter (if applicable).

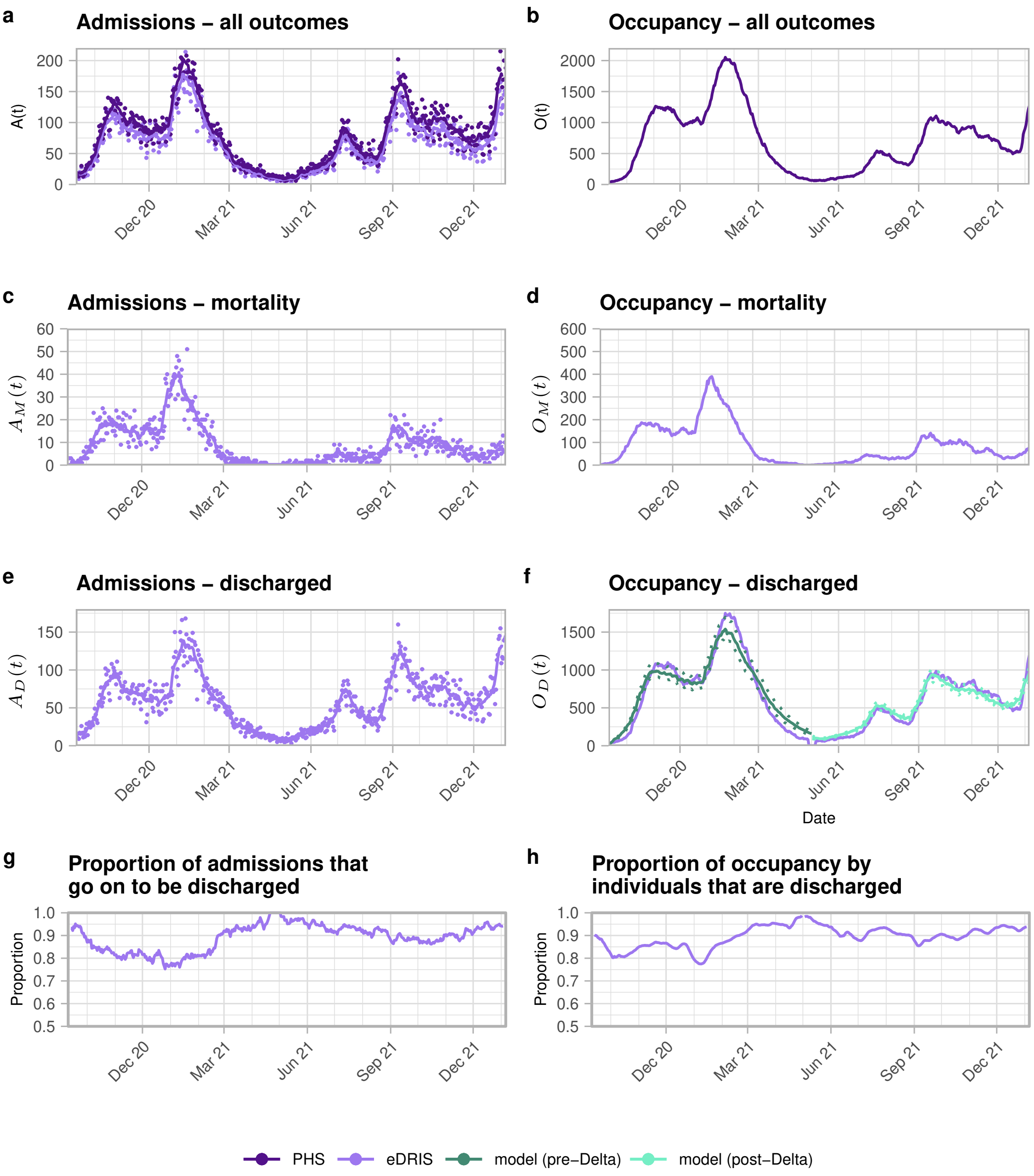

Figure 2: Trajectories of hospital admissions, and occupancy, of COVID-19 patients in Scotland. Overall admissions (a) and occupancy (b) are from Public Health Scotland (darker purple). PHS-published admissions are generally higher than those derived from the eDRIS data stream (lighter magenta). We then infer which of those admissions were from patients that eventually died (c), and the hospital occupancy of those individuals (d). The remaining admissions (e) and occupancy (f) are then taken to be from patients that go on to be discharged (and we assume survive), with (f) also including the fit occupancy (green) of individuals that go on to be discharged from our inferred admission-to-discharge intervals. Finally, the proportion of admissions (g) and occupancy (h) by patient outcome.

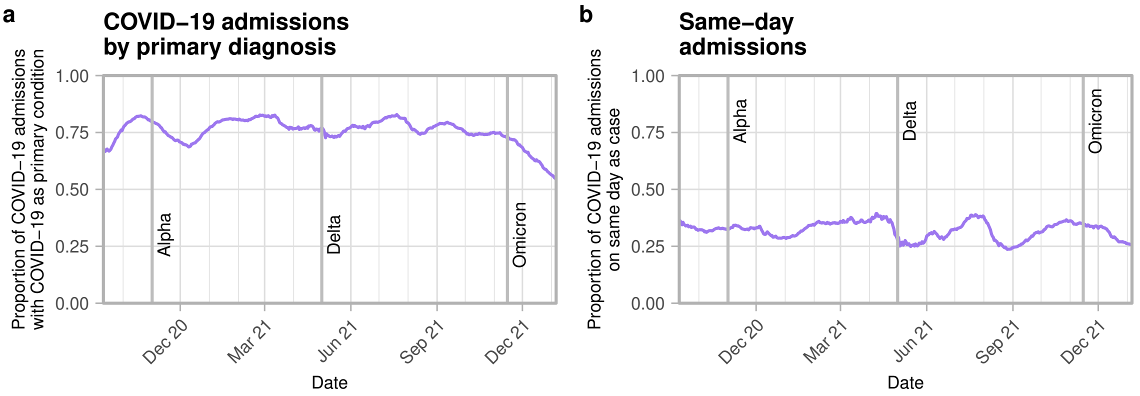

Figure 3: 28-day rolling mean of proportion of COVID-19 hospital admissions by (a) whether COVID-19 was the primary reason for admission, (b) whether the admission was on the same day as the related COVID-19 case (for admissions with an associated case). The approximate times of introduction of the Alpha, Delta and Omicron variants into Scotland (November 1 2020, May 1 2021 and November 15 2021 respectively) are marked.
