# Supplementary material for: Empirical distributions of time intervals between COVID-19 cases and more severe outcomes in Scotland
Source: PLoS One. 2023 Aug 16;18(8):e0287397. doi: 10.1371/journal.pone.0287397 (PMC10431635; doi:10.1371/journal.pone.0287397)
Supplement: S1 Appendix — (PDF) [file pone.0287397.s004.pdf]

S1 Appendix

Time intervals between COVID-19 cases and more severe outcomes; empirical distributions from Scottish data

Anthony J Wood, Rowland R Kao

A Associating outcomes

We define an interval $\Delta t_{\mathrm{AB}} \geq 0$ as the time difference between two different COVID-19 outcomes A and B, given as a whole number of days. We do not differentiate by any intermediate outcomes; for example, the case-to-mortality intervals includes both patients that were and were not admitted to an ICU.

We link different events with one another. For example, consider a hospitalisation entry H, for which we are attempting to associate a case C. To do this we:

1. Search for cases $\{C\}$ from the eDRIS test data, where the DZ, age range and sex matches with H, and occurred on the same day as, or up to 28 days before H.

2. If at least one ~~one~~ possible matching case is found, take the interval Δt_{CH} as the time difference between H and and the median date of the candidate cases $\{C\}$. Otherwise, label the hospitalisation entry H as *unlinked*.

We associate outcomes up to 21 days apart, with the exception of case-to-mortality intervals, where we search over 28 days. For case intervals, *unlinked* instances are reports of more severe outcomes, but without an associated prior case reported. For nosocomial intervals, unlinked outcomes are common (such as a mortality without an ICU admission), and are not counted.

Finally, in the data we omit events with incomplete age/sex/DZ entries (as we use these to associate different outcomes), as well as repeat admissions by the same individual within a window of 60 days, taking only the first admission.

B Distribution fits

For fitting the empirical distributions derived from the eDRIS data, we choose two-parameter *gamma* distributions $P(\Delta t)$:

$$P(\Delta t) = \lambda \, (\Delta t')^{\alpha-1} \, \mathrm{e}^{-\beta \Delta t'}, \tag{1}$$

for $0 \leq \Delta t \leq 21$ days (28 days for Δt_{CM}) and zero outside this range, with α determining the characteristic shape of the distribution for smaller Δt, and β determining the rate of exponential decay as Δt increases, and λ a normalising constant fixing $\int_0^\infty \mathrm{d}(\Delta t') P(\Delta t') = 1$. A gamma distribution allows us to flexibly fit distributions where the modal value is either at or away from zero days. We perform the fits using the *fitdist* function (version 1.1–8) in R (version 4.1.3).

To account for instances where the case and a more severe outcome are on the same day ($\Delta t = 0$), case intervals are fit across $\Delta t \geq 1$ only, with a *zero-inflation* ν fit separately, to reflect the proportion of all "same-day" events:

$$P(\Delta t) = \begin{cases} \nu & 0 \leq \Delta t \leq 1 \\ \lambda' \left(\Delta t'\right)^{\alpha-1} e^{-\beta \Delta t'} & 1 < \Delta t \leq 21 \quad (28 \text{ for } \Delta t_{\mathrm{CM}}) \end{cases} \tag{2}$$

where λ' here fixes $\int_1^\infty \mathrm{d}(\Delta t') P(\Delta t') = 1 - \nu$.

C Estimation of hospitalisation-to-discharge intervals

In this section we detail the method to estimate a distribution for the interval between hospitalisation and discharge, for patients presumed to not die in hospital. This is a much broader estimate across the whole population, as we do not have explicit times between admission and discharge. We instead rely on public, national-level occupancy data, provided by PHS.

For those admitted with COVID-19 that go on to die, we first use the eDRIS data (and associated intervals between hospital admission and death) to derive a partial occupancy timeseries. The difference between this occupancy and the *overall* PHS occupancy is then taken as the occupancy of admitted individuals that are discharged. Finally, knowing the admission dates of patients that go on to survive (i.e., do not have an associated death) from the eDRIS data, we estimate the hospitalisation-to-discharge interval distribution, and thus how much surviving individuals on average contribute to the hospitalisation occupancy burden.

Formally, the trajectory of hospital admissions $A(t)$ includes those that go on to be discharged (and we assume recover) $A_{\mathrm{D}}(t)$, and those that die in hospital $A_{\mathrm{M}}(t)$:

$$A(t) = A_{\mathrm{D}}(t) + A_{\mathrm{M}}(t) \ . \tag{3}$$

Similarly, the trajectory of COVID-19 hospital occupancy $O(t)$ includes the occupancy of those that go on to recover and be discharged $O_{\mathrm{D}}(t)$ and those that go on to die $O_{\mathrm{M}}(t)$:

$$O(t) = O_{\mathrm{D}}(t) + O_{\mathrm{M}}(t) \ . \tag{4}$$

To estimate the discrete distribution $P(\Delta t_{\mathrm{HD}})$ for the (H)ospital admission-to-(D)ischarge interval,

we first rewrite the occupancy of those eventually discharged

$$O_{\mathrm{D}}(t) \approx \sum_{t' \leq t} A_{\mathrm{D}}(t') \left(1 - \sum_{\Delta t_{\mathrm{HD}}=0}^{t-t'} P(\Delta t_{\mathrm{HD}}) \right) \tag{5}$$

writing $O_{\mathrm{D}}(t)$ as a *convolution* of the admissions trajectory $A_{\mathrm{D}}(t')$, and the proportion of individuals remaining in hospital after interval $t - t'$. $(1 - P(0))$ is then the proportion of patients that are in hospital for at least one full day. Knowing $O_{\mathrm{D}}(t)$ and $A_{\mathrm{D}}(t)$, what remains is to fit an appropriate $P\left(\Delta t_{\mathrm{HD}}\right)$.

We assume $P(\Delta t_{\mathrm{HD}})$ follows a zero-inflated exponential distribution, with the zero inflation accounting for individuals admitted, but discharged without an overnight stay. We use a standard Approximate Bayesian Computation (ABC) algorithm in a two-parameter space (exponential decay rate β, and zero-inflation ν), and take two different fits for the periods September 10 2020 – April 30 2021, and May 1 2021 – January 6 2022.

The prior (for ν: $U(0, 1)$ and for β: $U(0.05, 1)$), allows for any zero-inflation, and restricts the mean occupancy of those that stay at least one night between 1.1 and 20 days.

We build a posterior by accepting parameters that generate modelled occupancy trajectories $\tilde{O}_{\mathrm{D}}(t)$ that best fit $O_{\mathrm{D}}(t)$. We sample 10^6 different pairs of parameters, from which we take the $1,000$ that produce via Eq. (5) the timeseries $\tilde{O}^*_{\mathrm{D}}(t)$ that best fits $O_{\mathrm{D}}(t)$, minimising the sum of absolute residuals

$$\epsilon^* = \sum_t \left| \tilde{O}_{\mathrm{D}}(t) - O_{\mathrm{D}}(t) \right| \ . \tag{6}$$

Posterior distributions for β and ν, the mean hospitalisation-to-discharge interval and of the overall hospitalisation-to-discharge interval distribution. are presented in Fig A.

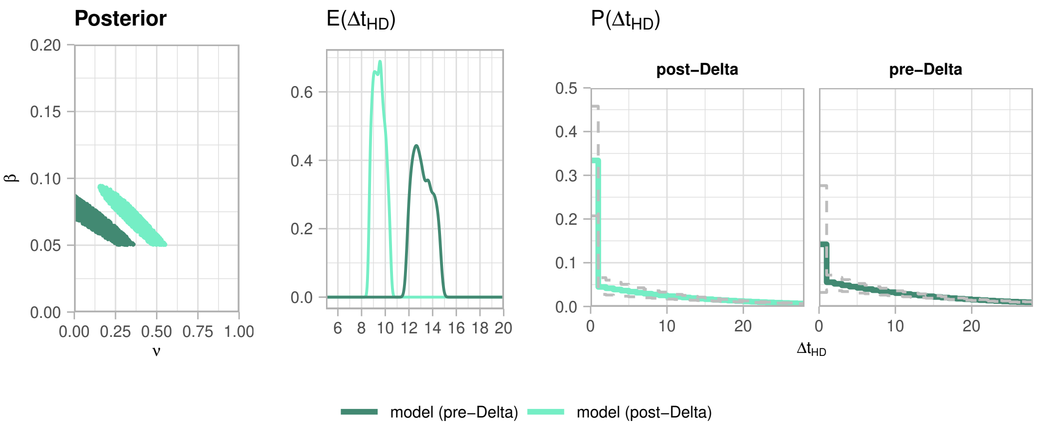

Figure A: Posterior distributions (left), mean intervals (centre) and distributions for hospitalisation-to-discharge intervals Δt_{HD}, across the "pre-Delta" (10 September 2020 – 30 April 2021) period, and "post-Delta" period (1 May 2021 – 6 January 2022) (right).
