# Supplementary material for: Empirical distributions of time intervals between COVID-19 cases and more severe outcomes in Scotland
Source: PLoS One. 2023 Aug 16;18(8):e0287397. doi: 10.1371/journal.pone.0287397 (PMC10431635; doi:10.1371/journal.pone.0287397)
Supplement: Attachment — Submitted filename: ReviewerLetter.pdf [file pone.0287397.s005.pdf]

Empirical distributions of time intervals between COVID-19 cases and more severe outcomes in Scotland

Rebuttal letter – feedback from reviewers: May 9 2023 (first received Apr 4 2023)

Anthony J Wood, Rowland R Kao

Reviewer 1

This paper presents an analysis of Covid-19-related hospitalization data in Scotland, between September 2020 and January 2022. Two different data sets are used and combined to estimate the distributions of time between given events in disease history: positive test, hospitalization, ICU admission and death. Anonymized patient-level data including age, sex and deprivation level of the area of residence were obtained from the Scottish electronic database.

Overall, this paper presents interesting results using data collected in a period of intense scrutiny, which will be useful in the future to fit models for Covid-19 outbreaks in regions that are less well-sampled. I believe that this paper can be published in Plos ONE. Below are some minor comments that might improve the presentation.

1. In the introduction, you mention variance of the interval distributions as a key feature related to the way in which hospital occupancy peaks are drawn out over time with respect to incidence peaks. This is important and justifies the fact that you are fitting distributions instead of comparing mean (or median) times. Perhaps you could add the variance estimates to Tables 1 and 2, as well as a brief discussion of variance in the Results or Discussion sections? The confidence intervals are not very informative in this case. Reply: We agree with the referee on the point that standard deviation is more informative than 90% CI's in this instance, and have now calculated standard deviations of the distributions in Tables 1 and 2. We also refer to standard deviation rather than CI's in the main text, and have added some discussion on variance (and the lack of variation-in-variance).

2. In a similar vein, confidence intervals could be added to the graphs in Fig. 2: one would assume that in the post-vaccination lull in the summer of 2021, low patient numbers lead to higher estimation error, which could explain the sudden jumps in t_CM and t_HM with the arrival of the Delta variant (or maybe this is a genuine effect related to epidemic dynamics?) In any case, it could be explored further, especially if these methods are to be applied to future real-time data, to prevent such artifacts from being mistaken as real shifts in the underlying interval distribution. Reply: Looking into this further, as the referee suggests the sudden jumps are likely attributable to these periods having few data points: for example, in May 2021 we have ∼20 Case-to-Mortality intervals, compared to ∼200 in February 2021. We agree it should be explained, however, and have updated this plot to show the standard error of the mean in the same rolling 28-day window (standard deviation / n(samples)), showing higher noise in the low-data period, and discussed these fluctuations in the main text.

3. Regarding the distribution fits of Fig. 1, it would be useful to add a few words on the method used. Also, why was a Gamma distribution chosen over e.g. a lognormal distribution? Reply: On the method, we have added some further description to the main text to supplement the more precise description in Appendix B. The main reason for choosing a gamma distribution is that a two-parameter fit felt appropriate (to capture differences in mean and characteristic variation) is that its parameter range allows for distributions where the peak sits away from zero (as most of the distributions have), or at zero (as seen in e.g., the hospital admission-to-ICU interval, where the zero peak is not a zero-inflation artefact). Whereas a lognormal distribution must have a value of zero, at zero. Now the standard deviation has been included alongside other measures, we hope that the distributions are well described now for a reader looking to use them.

4. Some typos:

   - p. 5: define the abbreviation "DZ" as it's the first tieme it is used in the text. Reply: DZ is now defined on the first use of the abbreviation (this arose due to moving the *Materials and Methods* section.

   - p.6, Fig.1: "(H)ospial" should be "(H)ospital" Reply: Typo has been corrected.

   - p.17, "If at least one one possible..." should be "If at least one possible..." Reply: Extra word has been removed.

Reviewer 2

The authors accessed outcome data from Public Health Scotland and collected publicly available data of deprivation data and nationwide-level occupancy of patients with COVID-19. They aimed to present a method for obtaining empirical distributions using COVID-19 outcomes data from Scotland between September 2020 and January 2022.

The authors report separate distributions for individual age, sex, and deprivation of residing community. They observed that the risk of severe disease following COVID-19 is higher for the elderly or those who reside in areas of high deprivation, the length of stay shows no strong dependence, suggesting that severe outcomes are equally severe across the risk groups. This study is likely to contribute to public health decision-making (or say will support future analysis of the disease, such as modelling and simulation). It is, therefore, worthy of publication, and the analysis is reasonably well-supported. However, I find some minor points that need to be addressed before publication can occur.

Minor comments:

1. The Appendix D (Estimation of hospitalisation-to-discharge intervals) seem better suited for the main text methods, contingent on the editor's approval. Reply: On reflection, the reviewer is right to highlight that the hospitalisation-to-discharge intervals methodology is out of place (that is, only mentioned in the appendix and outwith the *Materials and Methods*). As we see this part of the manuscript as more ancillary (the main emphasis of the manuscript being the use of high resolution data, whereas the inference is a much broader estimate with lower-resolution data) we feel it is appropriate to reserve the full details of the inference methodology for the appendix (as was done with the empirical work), but we have moved a summary of the methodology from the *Estimated hospitalisation-to-discharge interval* section to *Materials and Methods*, and moved a paragraph discussing possible sources of error from the Appendix to the *Results* section. This should give the reader a better understanding of the methodology than before, without needing to refer to the Appendix.

2. I recommend changing the colour of the figures to a colour-blind-friendly palette. Reply: We have updated this and now sample colours from the Okabe-Ito palette.

3. I suggest that the authors change the manuscript title to describe the study's locality. Reply: To reflect this we have changed the manuscript title to "*Empirical distributions of time intervals between COVID-19 cases and more severe outcomes in Scotland*".

4. Better present tables 1 and 2. The captions of both tables must be improved. "Case intervals" and "Nosocomical intervals" are not entirely describing what is in the tables Reply: We have added further explanatory text for both captions so the tables are understandable as stand-alone.

5. It would be good to describe the study limitations, if any. Reply: We have added a paragraph to the *Discussion* section outlining some limitations of the study.
